# Changes in the regulation of the Notch signaling pathway are temporally correlated with regenerative failure in the mouse cochlea

**DOI:** 10.3389/fncel.2015.00110

**Published:** 2015-03-31

**Authors:** Juan C. Maass, Rende Gu, Martin L. Basch, Joerg Waldhaus, Eduardo Martin Lopez, Anping Xia, John S. Oghalai, Stefan Heller, Andrew K. Groves

**Affiliations:** ^1^Department of Neuroscience, Baylor College of MedicineHouston, TX, USA; ^2^Department of Otolaryngology, Hospital Clínico Universidad de ChileSantiago, Chile; ^3^Interdisciplinary Program of Physiology and Biophysics, ICBM Universidad de ChileSantiago, Chile; ^4^Department of Otolaryngology, Clínica Alemana de Santiago, Facultad de Medicina Clínica Alemana-Universidad del DesarrolloSantiago, Chile; ^5^Department of Otolaryngology — Head and Neck Surgery, Stanford University School of MedicinePalo Alto, CA, USA; ^6^Department of Molecular and Human Genetics, Baylor College of MedicineHouston, TX, USA; ^7^Program in Developmental Biology, Baylor College of MedicineHouston, TX, USA

**Keywords:** notch, hair cell, supporting cell, cochlea, regeneration

## Abstract

Sensorineural hearing loss is most commonly caused by the death of hair cells in the organ of Corti, and once lost, mammalian hair cells do not regenerate. In contrast, other vertebrates such as birds can regenerate hair cells by stimulating division and differentiation of neighboring supporting cells. We currently know little of the genetic networks which become active in supporting cells when hair cells die and that are activated in experimental models of hair cell regeneration. Several studies have shown that neonatal mammalian cochlear supporting cells are able to trans-differentiate into hair cells when cultured in conditions in which the Notch signaling pathway is blocked. We now show that the ability of cochlear supporting cells to trans-differentiate declines precipitously after birth, such that supporting cells from six-day-old mouse cochlea are entirely unresponsive to a blockade of the Notch pathway. We show that this trend is seen regardless of whether the Notch pathway is blocked with gamma secretase inhibitors, or by antibodies against the Notch1 receptor, suggesting that the action of gamma secretase inhibitors on neonatal supporting cells is likely to be by inhibiting Notch receptor cleavage. The loss of responsiveness to inhibition of the Notch pathway in the first postnatal week is due in part to a down-regulation of Notch receptors and ligands, and we show that this down-regulation persists in the adult animal, even under conditions of noise damage. Our data suggest that the Notch pathway is used to establish the repeating pattern of hair cells and supporting cells in the organ of Corti, but is not required to maintain this cellular mosaic once the production of hair cells and supporting cells is completed. Our results have implications for the proposed used of Notch pathway inhibitors in hearing restoration therapies.

## Introduction

The Notch signaling pathway is an evolutionarily ancient form of cell-cell communication. During Notch signaling, the binding of membrane-bound ligands of the Delta and Jagged/Serrate families to Notch receptors causes the cleavage of the receptor and release of an intracellular domain which travels to the nucleus and participates in transcriptional activation (Artavanis-Tsakonas et al., 1999; Ilagan and Kopan, 2007; Artavanis-Tsakonas and Muskavitch, 2010; Hori et al., 2013). Notch signaling is deployed in the development of many tissues, and can influence cell fate through lateral inhibition with feedback (Chitnis, 1995; Formosa-Jordan et al., 2013), inductive signaling (frequently to establish boundaries of different cell types) or by the asymmetrical inheritance of fate determinants that affect Notch signaling (Bray, 2006).

Notch signaling regulates many aspects of inner ear development (Kelley, 2003, 2006, 2007; Murata et al., 2012). During the induction of the otic placode, the anlagen of the inner ear, Jagged1 activation of Notch signaling acts to strengthen otic placode fate in response to FGF and Wnt signals (Jayasena et al., 2008; Groves and Fekete, 2012). As the first neuroblasts begin to differentiate and delaminate from the otocyst, Notch-Delta signaling regulates the proportion of progenitor cells that differentiate as neurons by lateral inhibition (Adam et al., 1998; Abelló et al., 2007; Daudet et al., 2007; Kiernan, 2013; Neves et al., 2013a). As the first sensory regions of the inner ear begin to develop, Notch-Jagged1 signaling helps maintain and promote the fate of vestibular sensory regions of the ear through lateral induction (Eddison et al., 2000; Daudet and Lewis, 2005; Kiernan et al., 2005a; Brooker et al., 2006; Daudet et al., 2007; Hartman et al., 2010; Pan et al., 2010, 2013; Neves et al., 2011, 2013a,b), although it is less clear if this mode of sensory induction also occurs in the cochlea (Basch et al., 2011; Yamamoto et al., 2011). Finally, as hair cell and supporting cells begin to differentiate from sensory progenitor cells in prosensory patches, hair cells begin to express the Notch ligands Delta1 and Jagged2 (Dll1, Jag2) on their cell surface, and signaling by these ligands through the Notch1 receptor on nascent supporting cells induces and maintains supporting cell fate though lateral inhibition. Accordingly, pharmacological or genetic disruption of Notch1, Dll1 or Jag2, singly or in combination, leads to a failure of Notch signaling and an increase in the number of hair cells at the expense of supporting cells, likely through loss of lateral inhibition (Kiernan et al., 2005a; Brooker et al., 2006). Mutation or knock-down of downstream transcriptional effectors of Notch signaling, such as members of the Hes and Hey gene families, also leads to an increase in hair cell numbers at the expense of supporting cells (Zheng et al., 2000; Zine et al., 2001; Hayashi et al., 2008; Li et al., 2008; Doetzlhofer et al., 2009; Tateya et al., 2011; Benito-Gonzalez and Doetzlhofer, 2014).

A number of studies suggest that Notch signaling between hair cells and supporting cells continues in the sensory end organs of the mammalian inner ear after birth (Zine et al., 2000; Murata et al., 2006; Yamamoto et al., 2006; Hartman et al., 2007, 2009; Hori et al., 2007; Batts et al., 2009; Doetzlhofer et al., 2009; Lin et al., 2011; Liu et al., 2012a,b). Downstream effectors of Notch signaling can be detected in the cristae and maculae of the vestibular system (Hartman et al., 2009; Wang et al., 2010; Lin et al., 2011; Slowik and Bermingham-McDonogh, 2013), and blockade of Notch signaling in the adult vestibular system can induce the formation of ectopic hair cells at the expense of supporting cells (Lin et al., 2011; Slowik and Bermingham-McDonogh, 2013). Similarly, inhibition of Notch signaling in the neonatal organ of Corti also down-regulates some downstream effectors of Notch signaling in supporting cells and leads to the rapid formation of extra hair cells (Doetzlhofer et al., 2009; Korrapati et al., 2013; Mizutari et al., 2013; Bramhall et al., 2014). In both the neonatal cochlea and adult vestibular system, the generation of hair cells has been proposed to occur through a direct trans-differentiation of supporting cells without cell division (Doetzlhofer et al., 2009; Lin et al., 2011; Bramhall et al., 2014), a mode of differentiation that has also been observed during hair cell regeneration in birds (Stone and Cotanche, 2007).

Recently, application of gamma secretase inhibitors that attenuate the Notch pathway to the noise-damaged cochlea has been shown to generate small numbers of new hair cells and a partial restoration of hearing (Mizutari et al., 2013), suggesting that the Notch pathway may still be active in the mature cochlea. However, two issues remain unaddressed by this study. First, it is not clear to what extent components of the Notch signaling pathway—Notch receptors, Notch ligands and their downstream effectors—are expressed in the maturing cochlea (Batts et al., 2009). Indeed, two studies examining the presence of the active cleaved intracellular portion of the Notch1 receptor found very little evidence for Notch activation in the cochlea 1 week after birth (Murata et al., 2006; Liu et al., 2013). Moreover, although gamma secretase inhibitors are known to inhibit cleavage and activation of Notch receptors, they also cleave many other membrane proteins, and so it is possible that their effects in the cochlea may not be specific to the Notch pathway (Kopan and Ilagan, 2004).

In the present study, we compared the effects of gamma secretase inhibitors or blocking antibodies to the Notch1 receptor on the patterning of hair cells and supporting cells in organ cultures of the neonatal cochlea. In each case, we found that inhibitor treatment causes an increase in hair cell numbers at the expense of supporting cells, suggesting that both inhibitors are likely causing supporting cell trans-differentiation through inhibition of the Notch pathway. However, we find a precipitous age-dependent decline in the ability of these inhibitors to cause supporting cell trans-differentiation into hair cells. This decline in response to Notch inhibition progresses in a basal-apical gradient along the organ of Corti, consistent with the gradient of cellular maturation in the cochlea, and by 6 days after birth, the organ of Corti is essentially unresponsive to Notch inhibition in culture. We combined *in situ* hybridization, Q-PCR quantitation and single cell Fluidigm analysis of Notch pathway components and showed that Notch receptors, ligands and effectors are down-regulated from the organ of Corti in basal-apical gradient during the first postnatal week, and are at least an order of magnitude lower in mature animals, even after noise damage. Our results suggest that the Notch signaling pathway is deployed to establish the pattern of hair cells and supporting cells in the cochlea, but is not required to maintain this pattern in the mature organ of Corti.

## Materials and Methods

### Mice

*Atoh1*^A1GFP/A1GFP^ (MGI: *Atoh1*^tm4.1Hzo^) knock-in mice and *Atoh1*^GFP^ transgenic reporter mice were generated as previously described (Lumpkin et al., 2003; Rose et al., 2009). ICR mice were used for Notch Intracellular Domain (NICD) immunostaining. Noise damage experiments were performed on wild type CBA/CaJ mice. Pillar cells and Deiters’ cells were purified from *Fgfr3-iCreER*^T2^ (Young et al., 2010) mice mated with *Ai14:Rosa*^tdTom^ reporter mice (Jackson, #007908). All animal experiments were approved by the Baylor College of Medicine or Stanford University Institutional Animal Care and Use committees.

### Cochlear Organ Culture

Cochleas were dissected in ice cold HBSS immediately after euthanasia. Briefly, the heads were bisected, the temporal bone was removed from the skull base and the otic capsule was removed with forceps until the intact membranous cochlea was separated from the bony structures. For P0 and P3 animals, the cochlear duct was peeled out from the modiolus and the medial structures (Kölliker and Corti’s organs) were separated from the lateral wall, Reissner’s membrane and the stria vascularis. For P6 mice, in order to preserve the structures in the organ of Corti, the cochlear duct was gently separated from the modiolus by cutting between them with forceps and the lateral wall, stria vascularis and Reissner’s membrane were partially removed after cutting with 27 gauge needle. Immediately after dissection, the explants were placed on top of filter membranes with 1 μm pores (SPI-pore or Whatman) floating in DMEM/F12 (Hepes) supplemented with B27 supplements (Life Technologies), 1mM N-acetylcysteine (Sigma), 5 ng/ml EGF and 2.5 ng/ml FGF2 and 67 μg/ml penicillin. In some cultures DMEM/F12 medium was supplemented with N2 supplements (Life Technologies), N-acetylcysteine and penicillin. For Notch inhibition experiments, cultures were supplemented with 0.75–10 μM DAPT (Gamma secretase inhibitor IX, Calbiochem EMD) or DMSO 0.04% v/v (Life Technologies). Anti Notch1-specific antibodies and control IgD antibodies (Wu et al., 2010) were provided by Genentech and used at 2 μg/ml. The cultures were maintained for 1 h, 1, 2 or 3 days *in vitro* (DIV) at 37°C in 5% CO_2_.

### Immunostaining, Microscopy and Quantification

Whole cochlear explants were fixed in 4% paraformaldehyde, then permeabilized and blocked in 0.2% Triton X-100 and 10% donkey serum in PBS. The explants were incubated with primary antibodies overnight at 4°C, washed in PBST (0.1% Triton X-100 in PBS) and incubated with secondary antibodies for 2 h at room temperature, followed by 3 further washes in PBST and then incubated in 10 μg/ml DAPI for 10 min. Primary antibodies used were rabbit polyclonal anti-Myosin VIIa (1:500; Proteus Biosciences) and mouse monoclonal anti-GFP (1:200; Invitrogen). Secondary antibodies were Alexa Fluor 594 and 488 (Invitrogen). Images were obtained on an Axio Observer Zeiss microscope with an Apotome2 structured illumination attachment and analyzed in Axiovision 4.8 (Zeiss) and Image J (NIH) using Bioformat and the Cell Counter plugins. The cochlea was divided in five pieces: the tip, the apex, the middle, the base and the hook, and the center of each portion was analyzed, discarding the tip and the hook. Cell counts across different areas of the cochlea were normalized to cells per 100 μm and were expressed as a percentage increase with respect to control conditions. Significant differences were analyzed using a Mann-Whitney test for pairwise comparisons.

For NICD immunostaining in sections the protocol was modified from Morimoto et al. (2010). Briefly the heads of P0, P3 and P6 ICR mice were mounted in paraffin blocks and sectioned at 10 μm. The sections were rehydrated, bleached in H_2_O_2_ and boiled in a pressure cooker in antigen unmasking solution (Vectorlabs) for 20 min. The sections were then permeabilized, blocked and incubated with a cleaved Notch1 antibody (val1744; 1:100; Cell signaling) and anti- Myosin VIIa (1:500; Proteus Biosciences). The signal was amplified with Vectastain ABC Kit (rabbit IgG) (Vectorlabs) as indicated by manufacturer. The color was developed for 5–10 min with TSA Tyramide Cy3 Reagent (diluted 1:100 after reconstitution; Perkin Elmer) and then stained with DAPI. Images were obtained on a Zeiss Axio Observer microscope with an Apotome2 structured illumination attachment.

### *In Situ* Hybridization

Digoxygenin-labeled *in situ* probe synthesis was performed on linearized plasmid DNA using standard protocols (Stern, 1998).The following mouse cDNA probes were used in the study and kindly provided by the investigators listed: *Notch1, Jag1, Dll1* (Gerry Weinmaster), *Notch3* (Urban Lendahl), *Hes5* (Ryoichiro Kageyama), *Hey1, Hey2, HeyL* (Manfred Gessler), and *Atoh1* (Huda Zoghbi). A cDNA clone for *Jag2* (BC010982) was purchased from open Biosystems. The *in situ* hybridization procedure for frozen sections was modified from previous protocols (Harland, 1991; Birren et al., 1993; Groves et al., 1995). Heads of perinatal mouse pups were fixed in 4% paraformaldehyde in PBS overnight at 4°C, cryoprotected in 30% sucrose in PBS at 4°C, embedded in OCT compound (Sakura Finetek), and cryosectioned at 14 μm. Sections were fixed in 4% paraformaldehyde in PBS, pH 7.2 for 10 min at room temperature, followed by three 5-min washes in DEPC-treated PBS. The sections were treated with 1 μg/ml Proteinase K in DEPC-PBS for 5 min at room temperature, followed by three 5-min washes in DEPC-PBS and re-fixation in 4% paraformaldehyde in PBS, pH 7.2 for 10 min at room temperature. Sections were acetylated in 0.25% acetic anhydride in 0.1 M triethanolamine, pH 8.0 for 10 min at room temperature, followed by three 5-min washes in DEPC-PBS. Slides were incubated in hybridization buffer (50% formamide, 5 × SSC, 50 μg/ml Yeast tRNA, 100 μg/ml Heparin, 1 × Denhardt’s Solution, 0.1% Tween 20, 0.1% CHAPS, 5 mM EDTA) for 1–2 h at 65°C. 100 μl of digoxygenin-labeled probe (1 mg/ml) was added to each slide and the slides covered with glass coverslips. The slides were incubated in a chamber humidified with 5 × SSC, 50% formamide at 65°C overnight. Coverslips were removed by rinsing in 0.2 × SSC and the slides washed in 0.2 × SSC at 65°C for 1 h. The slides were then washed in 0.2 × SSC for 5 min at room temperature, followed by another 5-min wash in 0.1% Tween-20 in PBS (PTw). The slides were blocked in 10% lamb serum in PTw at room temperature for 1 h and then stained with anti-digoxygenin-alkaline phosphatase antibody (1:2000) for 1–3 h at room temperature in a humidified chamber. The slides were then washed three times for 5 min each in PTw and equilibrated with freshly-made alkaline phosphatase buffer (100 mM Tris pH 9.5, 50 mM MgCl_2_, 100 mM NaCl, 0.1% Tween 20) for 10 min. The slides were developed in alkaline phosphatase buffer containing 0.33 mg/ml NBT and 0.18 mg/ml BCIP in the dark at room temperature until the purple reaction product had developed to a satisfactory degree. The reaction was stopped by washing the slides in PBS three times for 15 min each, followed by fixation in 4% paraformaldehyde in PBS, pH 7.2 for 30 min. The slides were then rinsed and mounted in 80% glycerol in PBS. Whole mount *in situ* hybridization was carried out as recently described in detail (Khatri and Groves, 2013).

### RNA Isolation and Q-PCR

For each experimental condition, total RNA was extracted from 4 uncultured whole cochlear explants or 3 cultured cochlear explants using the RNeasy mini kit (Qiagen). RNA yield ranged from 480 to 1200 ng and was used for cDNA preparation using random primers and SuperScript III First-Strand Synthesis System (Invitrogen). qPCR reaction was performed with SYBRGreen PCR Master Mix in StepOnePlus RealTime PCR System (Applied Biosystems), using in the reaction cDNA at 0.3–0.6 ng/μl and primers at 50 nM (excepting 100 nM for the Hes5 reaction). Primers sequences are provided in Table 1. *GAPDH* and *L19* primers were used as reference genes. Significant differences were analyzed using a Mann-Whitney test for pairwise comparisons. Multiple comparisons or pairwise correction for multiple comparisons were not performed.

**Table 1:** **Q-PCR primers for Figures 1, 3, 5, 7**.

Gene	Forward primer	Reverse primer
Atoh1	5′-ATGCACGGGCTGAACCA-3’	5′-TCGTTGTTGAAGGAC
		GGGATA-3′
Notch1	5′-GCCGCAAGAGGCTTGAGAT-3′	5′-GGAGTCCTGGCATC
		GTTGG-3′
Notch3	5′-GCACTTGCCGTGGTTACATG-3′	5′-CCTCACAACTGTCACCAGC
		ATAG-3′
Hey1	5′-CACTGCAGGAGGGAAAGGTTAT-3′	5′-CCCCAAACTCCGATAG
		TCCAT-3′
Hey2	5′-AAGCGCCCTTGTGAGGAAA-3′	5′-TCGCTCCCCACGT
		CGAT-3′
HeyL	5′-GCGCAGAGGGATCATAGAGAA-3′	5′-TCGCAATTCAGAAAGGC
		TACTG-3′
Hes5	5′-GCACCAGCCCAACTCCAA-3′	5′-GGCGAAGGCTTTGC
		TGTGT-3′
Jag1	5′-AAAGACCACTGCCGTACCAC-3′	5′-GGGGACCACAGACG
		TTAGAA-3′
Jag2	5′-TGCGAACTAGAGTACGACAA-3′	5′-TTGGTTCACAGAGAT
		CCATG-3′
Dll1	5′-TCCGATTCCCCTTCGGCTTCA-3′	5′-TCTGTTGCGAGGTCA
		TCGGGA-3′
Dll3	5′-CCGCTTTCCCAGACGCTGAT-3′	5′-GGCCTGGCCCGAAA
		GAATGA-3′
Gapdh	5′-AGGTCGGTGTGAACGGATTTG-3′	5′-TGTAGACCATGTAGTTGA
		GGTCA-3′
Ll9	5′-GGTCTGGTTGGATCCCAATG-3′	5′-CCCGGGAATGGA
		CAGTCA-3′

### Single Cell Purification and Q-PCR Analysis

Triple transgenic mice heterozygote for Fgfr3-iCreER^T2^ (Young et al., 2010), Ai14:Rosa^tdTom^ (Jackson, #007908) and Sox2-EGFP (Jackson, #017592) were analyzed at P2, and double transgenic animals (Fgfr3-iCreER^T2^ and Ai14:Rosa^tdTom^) were analyzed at P21. Animals were injected with 0.2 mg/g body weight tamoxifen at P0 and P19 respectively. Animals were euthanized 2 days after the tamoxifen injection and organs of Corti were dissected. Single cell dissociation, flow cytometry, RNA isolation and single cell qRT-PCR were performed as described in Durruthy-Durruthy et al. (2014) using the primers listed in Table 2. Briefly, expression of Actb or Gapdh at levels lower or higher than 3 standard deviations from the mean was used to exclude compromised cells/empty wells or possible doublets, respectively. Ai14-Control primers detect recombination within the Ai14-tdTomato reporter locus and cells with no detectable recombination were excluded from the analysis. Single cell expression data is presented as Log2Ex values, calculated by subtracting experimentally determined Ct-values from the median limit of detection calculated for all primers used in the study. Single cell data were normalized using the median Log2Ex values as recommended by Fluidigm.

**Table 2:** **Q-PCR primers for Figure 6**.

Gene	Forward primer	Reverse primer
Actb:	5′- CCCTAAGGCCAACCGTGAAA -3′	5′- CAGCCTGGATG
		GCTACGTAC -3′
Gapdh	5′- AGACGGCCGCATCTTCTT -3′	5′- TTCACACCGACC
		TTCACCAT -3′
Ai9-Control	5′- AGGAACTTCGTCGACATTTAAATCA -3′	5′- CTGCAGGTCGA
		GGGACCTAA -3′
Fgfr3	5′- AGGATTTAGACCGCATCCTCAC -3′	5′- CCTGGCGAGTAC
		TGCTCAAA -3′
Cdkn1b	5′- CAGTGTCCAGGGATGAGGAA -3′	5′- TTCGGGGAACCGTC
		TGAAA -3′
Sox2	5′- TGAAGGAGCACCCGGATTATA -3′	5′- CGGGAAGCGTGT
		ACTTATCC -3′
Jag1	5′- AACGACCGTAATCGCATCGTA -3′	5′- TCCACCAGCAAAGT
		GTAGGAC -3′
Jag2	5′- CTCGTCGTCATTCCCTTTCA -3′	5′- GGTGTCATTGTC
		CCAGTCC -3′
Hes1	5′- TGAAGCACCTCCGGAACC -3′	5′- CGCGGTATTTCC
		CCAACAC -3′
Hes5	5′- AAGAGCCTGCACCAGGACTA -3′	5′- GTGCAGGGTCAGG
		AACTGTAC -3′
Hey1	5′- ACGAGACCATCGAGGTGGAA -3′	5′- CGTTGGGGACAT
		GGAACACA -3′
Hey2	5′- ACTAGTGCCAACAGCTTTTGAA -3′	5′- TGTAGCCTGGAGC
		ATCTTCA -3′
LFng	5′- TCGATCTGCTGTTCGAGACC -3′	5′- CCTCCCCATCAG
		TGAAGATGAA -3′

### Noise Damage

Noise damage of six-week-old CBA/CaJ mice was performed as previously described (Liu et al., 2011). Briefly, a custom-built box contained six piezo horns (TW-125, Pyramid Car Audio, Brooklyn, NY, USA) inserted through the cover. Band-passed white noise (4–22 kHz) was generated digitally with RPvds software (Version 6.6, Tucker-Davis Technologies, Alachua, FL, USA), converted to analog by a digital-to-analog converter, and then transferred to the power amplifier (Servo 550, Sampson, Hauppauge, NY, USA) to drive the speakers. A cage containing the mice was placed inside the box and the mice were exposed to noise at 98 dB ± 2 dB for 4 h.

## Results

### The Neonatal Cochlea Exhibits a Position-Dependent Variation of Supporting Cell Trans-Differentiation in Response to Gamma Secretase Inhibitors

**Figure 1:**
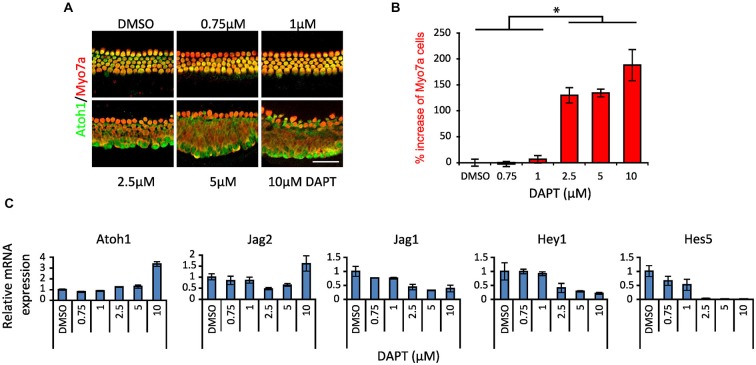
**Dose response of Notch inhibition in P0 cultures. (A)** Immunostaining of apical portions of cochlear explants of newborn (P0) *Atoh1*^A1GFP/A1GFP^ knock-in mice cultured 2 days *in vitro* (DIV) in DAPT from 0.75 to 10 μM or vehicle (DMSO). Atoh1: green. Myo7a: red. Scale 50 μm. **(B)** Quantitation of the increase in number of Myosin VIIa-labeled cells after different doses of DAPT compared to DMSO vehicle (same as shown in **A**). *N* = 4. *p = 0.030 (Mann-Whitney pairwise comparisons). Error bars: SEM. **(C)** mRNA amount of hair cell, supporting cell and Notch pathway genes obtained by QPCR in whole cochlear explants from P0 *Atoh1*^GFP^ transgenic reporter mice treated with DAPT compared to DMSO vehicle. *N* = 3. Error bars: SEM. Note that error bars are present for each condition but are very small in some cases.

A number of studies have demonstrated conversion or trans-differentiation of cochlear supporting cells into hair cells after treatment with gamma secretase inhibitors (Takebayashi et al., 2007; Hayashi et al., 2008; Doetzlhofer et al., 2009; Korrapati et al., 2013; Mizutari et al., 2013; Bramhall et al., 2014), although results in different studies have often been obtained with different gamma secretase inhibitors or at different concentrations of a given inhibitor. We confirmed these results in cochlear explant cultures from postnatal day 0 (P0) *Atoh1*^A1GFP/A1GFP^ and *Atoh1-GFP* mice, using the gamma secretase inhibitor DAPT over a range of 0.75–10 μM. After 2 days in culture, we stained the cultures for Myosin VIIa and GFP to reveal the Atoh1-GFP fusion protein (*Atoh1*^A1GFP/A1GFP^ mice) or GFP reporter (*Atoh1-GFP* mice). We observed a significant increase in the numbers of Myosin VIIa ^+^ hair cells at 2.5, 5 and 10 μM DAPT compared to DMSO vehicle (Figures 1A,B), but not at lower concentrations. We measured the levels of hair cell and supporting cell mRNAs in the *Atoh1-GFP* cultures and observed an increase of the hair cell markers *Atoh1* and *Jag2* and a decrease in the supporting cell markers *Jag1, Hey1* and *Hes5* (Figure 1C). As previously described (Doetzlhofer et al., 2009), *Hes5* was particularly sensitive to DAPT, with a strong reduction in mRNA levels being observed above 1 μM.

**Figure 2:**
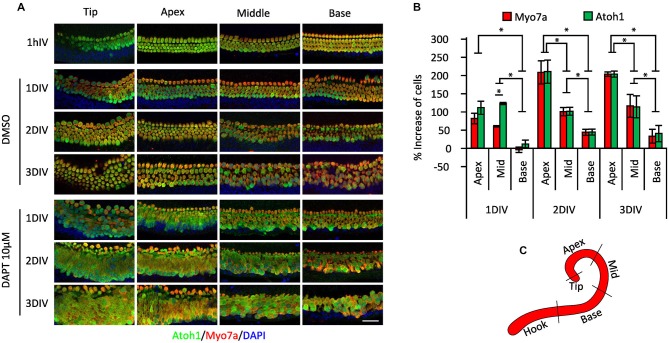
**Position-dependent effect of Notch inhibition in P0 cultures. (A)** Immunostaining of different cochlear portions from newborn (P0) *Atoh1*^A1GFP/A1GFP^ knock-in mice (shown in **C**) treated with 10 μM DAPT or vehicle (DMSO) for 1 h to 3 days *in vitro* (DIV). Atoh1: green. Myo7a: red. DAPI: Blue. **(B)** Percentage of increase in numbers of Myosin VIIa-labeled cells and GFP-labeled cells in the different regions of the cochlea after DAPT 10 μM treatment compared to vehicle (same as shown in **A**). *N* = 4. *p = 0.030 (Mann-Whitney pairwise comparisons). Error bars: SEM. **(C)** Schematic view of the cochlear portions evaluated in **(A,B)**.

The organ of Corti differentiates in a broadly basal-apical direction, with the first differentiating Atoh1^+^ hair cells appearing near the base of the cochlea, and a wave of hair cell and supporting cell differentiation spreading basally to the hook region and in an apical direction to the tip of the cochlea (Chen et al., 2002; Cai et al., 2013). Consequently at birth, hair cells and supporting cells in the basal region of the cochlea can be considered to be slightly more mature than their counterparts at the apex and tip. To determine whether these differences in maturity affected the response to gamma secretase inhibitors, we cultured whole P0 cochleas from Atoh1-GFP mice in 10 μM DAPT or DMSO vehicle for 1–3 days and counted the numbers of Atoh1-GFP or Myosin VIIa-expressing hair cells in the apical, middle and basal regions of the cochlea (Figures 2A,B; the approximate positions of the three regions are indicated in Figure 2C). We excluded from our counts the most basal hook region as it was more susceptible to variable damage during dissection and the most apical tip region because of its more variable behavior. We observed a clear effect of position on the number of supernumerary hair cells generated in the cultures over the 3 day culture period, with the apex producing 202% more hair cells after 3 days, whereas the base generated only 32% more hair cells in the same time period (Figures 2A,B). These results suggested that more mature supporting cells at the base of the cochlea were far less likely to trans-differentiate into hair cells in response to DAPT than their younger counterparts at the apex.

### The Response of Supporting Cells to Gamma Secretase Inhibition or Notch Inhibition Declines Rapidly with Age

**Figure 3:**
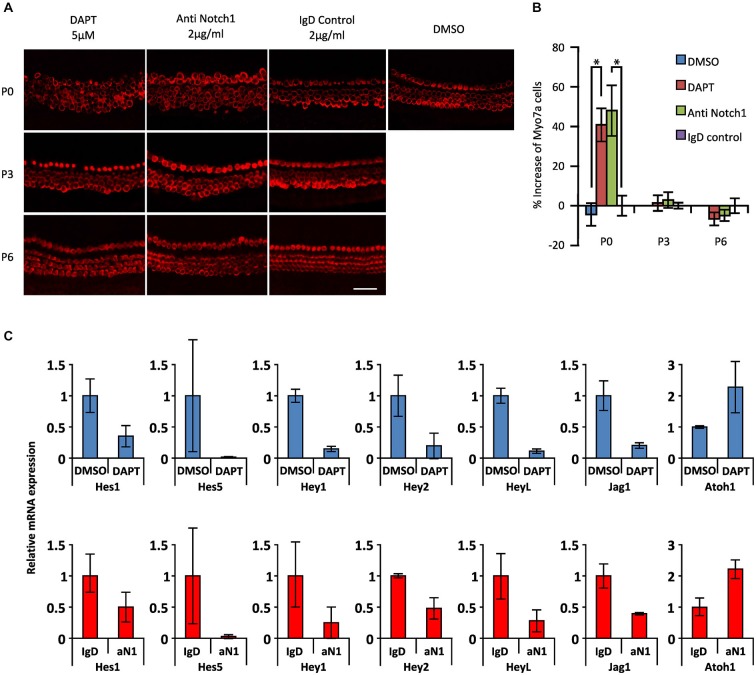
**Age-dependent decline in the effect of Notch inhibition on supporting cells in the presence of gamma secretase inhibitors or Notch1 blocking antibodies. (A)** Immunostaining of apical portions of cochlear explants obtained at 0, 3 and 6 postnatal days (P0, P3 and P6) from *Atoh1*^GFP^ transgenic reporter mice treated with 5 μM DAPT, 2 μg/ml Notch1 antibodies (Anti Notch1), 2 μg/ml control IgD and DMSO cultured 2 days *in vitro* (DIV). Myo7a: red. Scale 50 μm. **(B)** The increase in numbers of Myosin VIIa-labeled cells in the apical portion of the cochleas shown in **(A)**. *N* = 6, 6 and 3 for P0, P3 and P6 respectively. *p = 0.0044 and 0.0045 for DMSO/DAPT and Anti Notch1/Control IgD comparisons respectively (Mann Whitney). Error bars: SEM. **(C)** mRNA amount of Notch pathway genes obtained by QPCR in whole cochlear explants of *Atoh1*^GFP^ transgenic reporter P0 newborn mice cultured in the presence of DAPT or DMSO and in Notch1 antibodies or control IgD antibodies. Blue columns (top): level of expression after DAPT treatment relative to DMSO. Red columns (bottom): level of expression after anti Notch1 antibodies (aN1) relative to control IgD antibodies. *N* = 3. Error bars: SEM. Note that error bars are present for each condition but are very small in some cases.

To test whether the response of cochlear supporting cells to DAPT was indeed age-dependent, we established cochlear cultures from newborn (P0), 3 and 6 day old mice and cultured them for 2 days in 5 μM DAPT. We quantified the number of supernumerary hair cells in the apical region. We saw significant numbers of supernumerary hair cells in DAPT-treated P0 cochlear cultures compared to DMSO vehicle, but observed no significant increase in hair cell numbers when either P3 or P6 cultures were treated with DAPT (Figures 3A,B).

Most experiments in which gamma secretase inhibitors have been used to promote supporting cell trans-differentiation into hair cells are interpreted on the assumption that the inhibitors are targeting the cleavage of Notch receptors in cochlear supporting cells. However, since gamma secretases cleave many other membrane proteins (Kopan and Ilagan, 2004), it is possible that some of the effects of gamma secretase inhibitors may be due to the inhibition of cleavage of other membrane proteins. To test this, we used specific blocking antibodies to the Notch1 receptor (Wu et al., 2010) at a concentration of 2 μg/ml, previously shown to effectively inhibit Notch1 signaling *in vitro* (Wu et al., 2010). Cochlear cultures from P0 animals showed similar age-dependent responses to Notch1 blocking antibodies (48% increase in Myosin VIIa ^+^ hair cells) as cultures incubated for 2 days in 5 μM DAPT (41% increase) compared to control cultures containing DMSO or a control IgD (Figures 3A,B). However, we saw no significant response when P3 or P6 cultures were treated with blocking antibodies as we previously saw with 5 μM DAPT (Figures 3A,B). We also observed a comparable down-regulation of supporting cell-specific *Hes* and *Hey* genes and the supporting cell marker *Jag1* in P0 cultures treated with DAPT or Notch1 blocking antibodies, together with a comparable up-regulation of *Atoh1* (Figure 3C). These results suggest that the majority of the effects of the gamma secretase inhibitor DAPT on supporting cell trans-differentiation in neonatal cultures are likely specific to the Notch pathway.

### Notch Pathway Genes are Down-Regulated in the Cochlea During the First Postnatal Week

**Figure 4:**
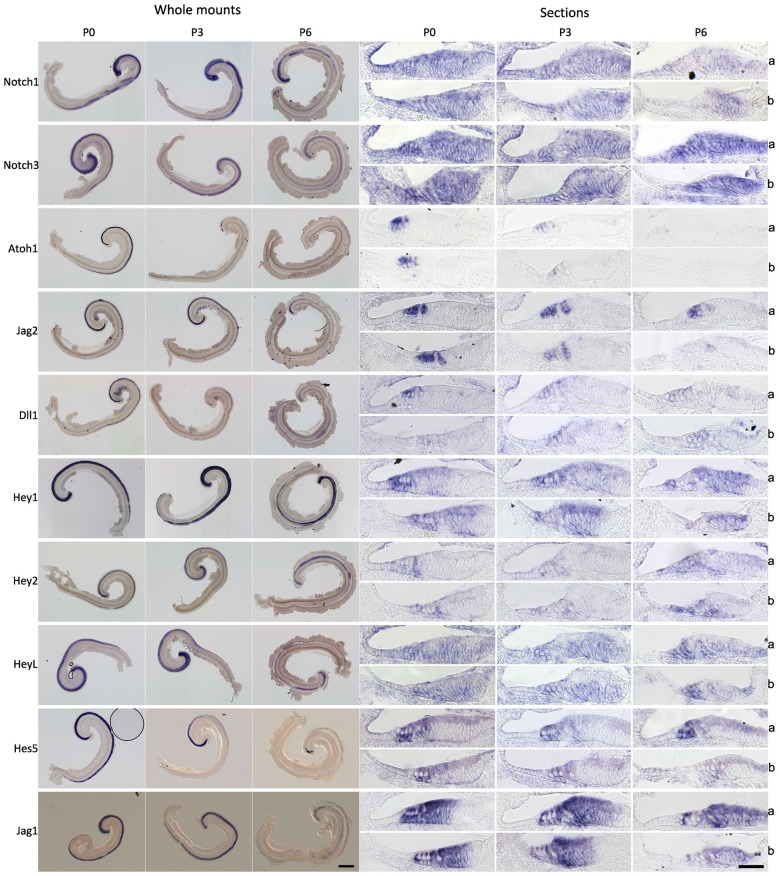
**Notch pathway components are down-regulated during the first postnatal week**. *In situ* hybridization of Notch pathway genes in the cochlea at 0, 3 and 6 postnatal days (P0, P3 and P6). Left panels: Whole mount *in situs* of cochlear explants, with the samples curved clockwise from apex to base. Scale = 200 μm. Right panels: *In situ* hybridization of frozen sections; a: apex region, b: basal region. Scale 50 μm.

**Figure 5:**
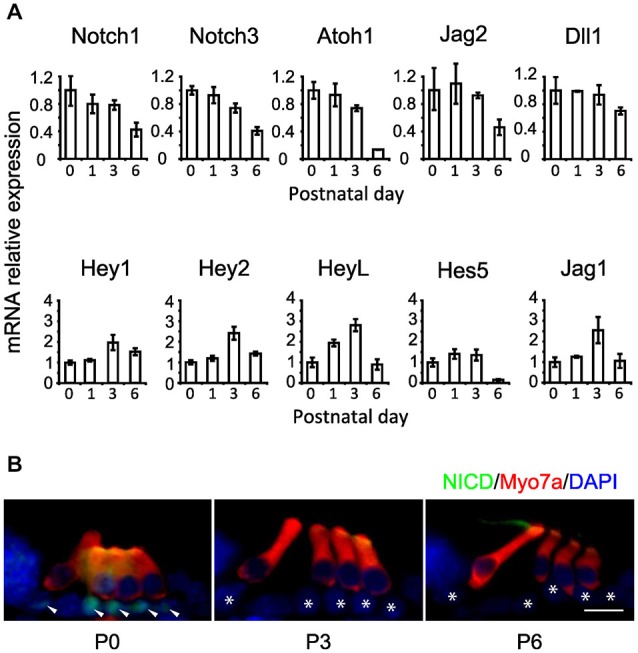
**Notch pathway components and Notch1 signaling activity decline during the first postnatal week. (A)** Relative expression of some Notch pathway genes obtained by QPCR from whole cochlear explants at 1, 3 and 6 postnatal days relative to newborn (0 postnatal days). *N* = 3. Error bars: SEM. Note that error bars are present for each condition but are very small in some cases. **(B)** Notch1 intracellular domain (NICD) immunostaining in cochlear sections of ICR newborn mice, obtained at 0, 3 and 6 postnatal days (P0, P3 and P6 respectively). NICD: green. Myo7a: red. DAPI: blue. Arrow heads: supporting cells positive for NICD staining. Stars: same supporting cells pointed by arrow heads but negative for NICD staining. Scale = 20 μm.

The preceding results suggest that the Notch pathway is deployed to stabilize supporting cell fate of neonatal cochlear supporting cells, but that inhibition of the Notch pathway has no effect on supporting cell fate even a few days later. To determine out if this change in the response of supporting cells to Notch inhibition was related to changes in the endogenous activity of the Notch pathway, we examined the expression of mRNA for Notch receptors (*Notch1* and *Notch3*), ligands (*Dll1, Jag1* and *Jag2*) and downstream effectors of Notch signaling (*Hey1, Hey2, HeyL* and *Hes5*) in the cochlea from P0 to P6 by *in situ* hybridization on whole mount cochleas, sectioned cochleas and by Q-PCR of cochlear tissue (Figures 4, 5A). In general, all components of the Notch pathway evinced a down-regulation between P0 and P6 starting at the base and proceeding down to the apex. Specifically, *Notch1* and *Notch3* were expressed throughout the supporting cell layer and into Kölliker’s organ and the outer sulcus, and both receptors showed a basal-apical down-regulation between P0 and P6. *Jag2* and *Dll1* were both down-regulated in hair cells between P0 and P6, along with the hair cell marker *Atoh1*. *Hey2* and *Hes5* were down-regulated from pillar cells and Deiters’ cells respectively in a basal-apical gradient, whereas *Hey1, HeyL* and *Jag1* were expressed in all supporting cells and cells of Kölliker’s organ, and down-regulated again in a basal-apical direction. The speed of down-regulation varied considerably from gene to gene—for example, *Dll1* was down-regulated in hair cells more quickly than *Jag2*, and *Hes5* was down-regulated much more quickly in supporting cells than *Hey1*. We also saw a general trend towards down-regulation of each gene by Q-PCR (Figure 5A), although the degree of down-regulation measured by this method was somewhat blunted as a result of including the entire basal-apical extent of the cochlear duct in each sample. To confirm that activation of the Notch1 receptor was also decreasing between P0 and P6, we immunostained cochlear sections with antibodies to the Notch1 intracellular domain (Notch1-ICD) which is released and localized to the nucleus after Notch activation (Figure 5B). We observed Notch1-ICD staining in Deiters’ cells at P0, but could not detect staining in the supporting cells at later stages.

**Figure 6:**
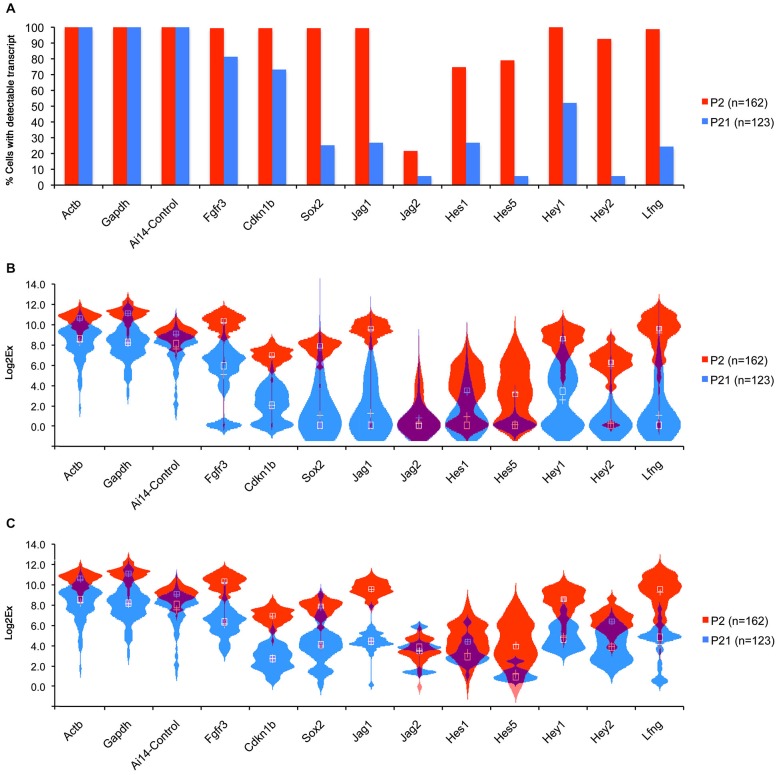
**Comparison of Notch pathway genes in P1 and P21 cochlea at the single cell level**. Pillar and Deiters’ cells were purified from P2 and P21 *FGFR3-CreER;ROSA-TdTomato* mice and RNA extracted from 162 (P2) and 123 (P21) single cells and subjected to QPCR analysis using the Fluidigm system (Durruthy-Durruthy et al., 2014) with primers for housekeeping genes and Notch pathway genes. **(A)** Graph showing the percentage of cells at each age that expressed detectable levels of each gene under analysis. **(B)** Violin plot showing the distribution of expression levels for each gene in all cells including the cells with no expression (Log2x = 0) presented in a combination of box plots and kernel density plots. White Crosses indicate the mean, white boxes the median expression levels. **(C)** Violin plot similar to **(B)**, excluding cells with undetectable levels of expression for each gene.

Although we did not see a significant increase in hair cells numbers at P3 or P6 after Notch inhibition in culture we did observe occasional isolated ectopic hair cells in our P3 (but not P6) cultures that may have been generated by trans-differentiation of supporting cells (Figures 3A,B) raising the possibility that a sub-population of supporting cells maintain Notch pathway expression at significant levels. To test whether small numbers of mature supporting cells maintain expression of some Notch pathway genes, we used the Fluidigm single cell handling system to compare gene expression in individual supporting cells purified from P2 and P21 mice. To label and purify pillar cells and Deiters’ cells at P2, we injected triple transgenic mice (*FGFR3-iCreERT2/Ai14:Rosa^tdTom^/Sox2-EGFP*) with tamoxifen at P0 and isolated TdTomato/EGFP double positive cells by flow cytometry. At this age, *FGFR3-iCreERT2* fate-labels pillar, Deiters’, and outer hair cells, whereas EGFP is confined to all supporting cells. At P21, we used double transgenic (*FGFR3-iCreERT2/Ai14:Rosa^tdTom^*) mice, injected with tamoxifen at P19, and sorted TdTomato-positive pillar and Deiters’ cells, which were the only organ of Corti cell types labeled at this age. cDNA was prepared from individual P2 (*N* = 162) and P21 (*n* = 123) TdTomato cells using the Fluidigm single cell analysis system, and 96 genes analyzed from each sample by Q-PCR, including the Notch pathway genes *Jag1* and *2, Hes1* and *5, Hey1* and *2*, and the Notch target and ligand modulator *LFng* (Figure 6). In all cases, the numbers of cells with detectable amounts of Notch pathway genes declined from P2 to P21, (Figure 6A). The distribution in expression levels of Notch pathway genes in individual cells was visualized in violin plots and revealed a clear downward shift in expression across the population from P2 to P21 (Figure 6B), even when cells with undetectable levels of expression were removed from the analysis (Figure 6C). In some cases, we saw evidence for a small population of cells expressing high levels of a single Notch pathway gene at P21 (e.g., *Jag2*; Figure 6C), but we were unable to observe any single cells at P21 that co-expressed high levels of multiple Notch genes. These data suggest that the majority of P21 *FGFR3-iCreERT2* fate labeled supporting cells are unlikely to be transducing significant Notch signaling.

### Notch Pathway Components are not Expressed at Significant Levels in the Normal and or Noise-Damaged Adult Organ of Corti

**Figure 7:**
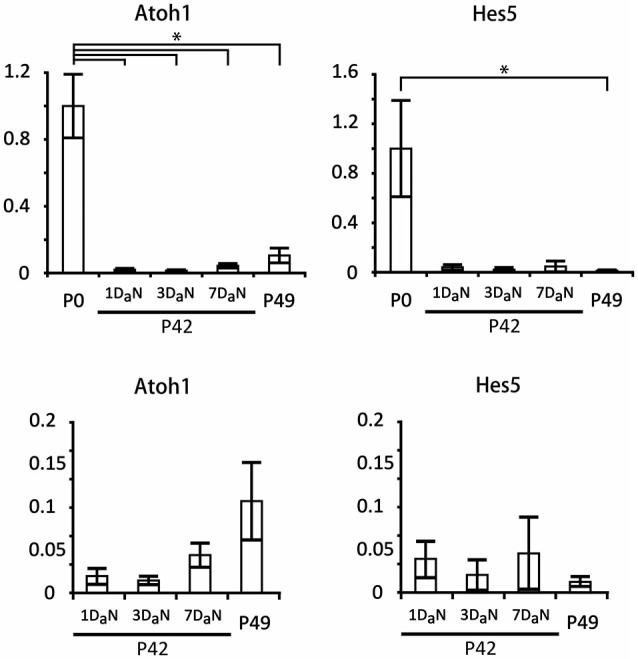
***Hes5* and *Atoh1* remain expressed at very low levels in the mature organ of Corti, even after noise damage**. Relative expression of *Atoh1* and *Hes5* obtained by QPCR in cochlear explants from neonatal (P0) and 6 to 7 week old mice exposed to noise. The mice exposed to noise on the 42nd postnatal day (P42) were evaluated after 1 (1D_a_N), 3 (3D_a_N), or 7 (7D_a_N), days. As controls for the 7 day cohort we used 7 week old mice (P49) that had never been exposed to noise. The expression levels of *Atoh1* and *Hes5* were normalized to the level of expression at P0. *N* = 3 in all cases except P0, where *N* = 6. The values for adult animals are re-plotted on separate graphs; note that no significant changes occur in the adult samples. Error bars: SEM. *p = 0.03689 (Mann-Whitney pairwise comparisons).

Several previous studies have suggested that some components of the Notch pathway may be re-activated in supporting cells following damage (Oesterle et al., 2008; Batts et al., 2009; Mizutari et al., 2013). However, these studies did not perform a direct quantitative comparison of message levels of Notch pathway components between neonatal animals (in which the Notch pathway is expressed and active) and mature animals before and after damage. We examined the expression of *Atoh1* and *Hes5* in cochleas isolated from cohorts of mice which received noise damage at P42 and were analyzed at one, three, or seven days later. Our controls were non-noise exposed cohorts of P0 and P49 mice. The level of noise we applied has been shown to be adequate to damage the cochlear epithelium and elicit changes in gene expression. In particular, it produces large temporary threshold elevations, and mild permanent threshold shifts, 17% OHC loss and 3% IHC loss, and increases in prestin gene expression in residual OHCs (Wang et al., 2010; Xia et al., 2013). We found that levels of the hair cell-specific transcription factor *Atoh1* in 7 week old animals were less than 10% of their neonatal counterparts (Figure 7) and that these levels did not change significantly over a 7 day period after noise damage. Similar results were observed for *Hes5* (Figure 7). These data suggest that the Notch pathway remains down-regulated in the mature cochlea and that it is not significantly perturbed by noise damage.

## Discussion

The Notch signaling pathway is deployed during the differentiation of hair cells and supporting cells and has been proposed to regulate the proportion of each cell type through lateral inhibition (Lewis, 1991; Eddison et al., 2000). The observation that inhibiting Notch signaling can generate ectopic hair cells at the expense of supporting cells (Takebayashi et al., 2007; Hayashi et al., 2008; Doetzlhofer et al., 2009; Korrapati et al., 2013; Mizutari et al., 2013; Bramhall et al., 2014), together with the observation that Notch signaling is re-deployed during avian hair cell regeneration (Stone and Rubel, 1999; Stone and Cotanche, 2007) has raised the possibility of targeting the Notch pathway in the damaged cochlea to effect hair cell replacement. However, results with Notch inhibitors in the adult cochlea have given variable results (Hori et al., 2007; Mizutari et al., 2013; Tona et al., 2014), prompting us to examine how this pathway is regulated as the cochlea matures. We show that the response of supporting cells to Notch inhibition drops dramatically in the first postnatal week, concomitant with a down-regulation of many components of the Notch signaling pathway.

Many studies have used gamma secretase inhibitors as a reagent to inhibit Notch signaling, despite the fact that gamma secretases are known to cleave scores of other transmembrane proteins in addition to Notch receptors (Kopan and Ilagan, 2004). Although it has generally been assumed that the conversion of supporting cells to hair cells in the presence of gamma secretase inhibitors is due to Notch inhibition, very few studies have tested this formally (Hayashi et al., 2008). We now show that the effects of the gamma secretase inhibitor DAPT on perinatal cochlear cultures—both in terms of the numbers of ectopic hair cells generated, in the down-regulation of Notch target genes and in the age-dependent response to these inhibitors—can be mimicked by blocking antibodies to the Notch1 receptor. While it remains formally possible that other gamma secretase-dependent signaling pathways are operating in perinatal supporting cells, our data suggest that the effect of inhibiting such pathways is negligible compared to their effect on Notch cleavage. We saw no evidence for supporting cell proliferation in our neonatal cultures treated with DAPT or Notch blocking antibodies (Doetzlhofer et al., 2009; data not shown). Conditional deletion of the Notch1 receptor, either at the otic placode stage (Kiernan et al., 2005a) or in neonatal mice (Li et al., 2015) has been reported to cause a small amount of supporting cell proliferation. Since these studies were both performed in intact animals, it is possible that the conditions used to establish organ cultures in our study militate against supporting cell proliferation when Notch signaling is blocked. Alternatively, it is possible that loss of a single allele in the *Sox2*-*CreER* and *Foxg1-Cre* knock-in lines used in these studies may interact genetically with *Notch1* mutants to cause abnormal proliferation. Indeed, haploinsufficiency of *Sox2* can modify the phenotype of *p27*^*Kip*1^ mutants (Li et al., 2012), and *Foxg1-Cre* knock-in mice have been shown to have brain defects associated with proliferative defects on certain genetic backgrounds (Shen et al., 2006; Eagleson et al., 2007; Siegenthaler et al., 2008; see Cox et al., 2012 for further discussion).

We have characterized an age-dependent decline in the response of cochlear supporting cells to Notch inhibition in two ways. Our results from the most direct test of such age-dependence—treating cochlear tissue of different ages with Notch inhibitors (Figure 3)—are also supported by a careful analysis of basal-apical differences in the response of neonatal cochlear cultures to Notch inhibitors (Figure 2). Since hair cells and supporting cells in the mid-base of the cochlea differentiate at least 3 days before cells at the apex (Chen et al., 2002; Cai et al., 2013), analysis of whole cochlear explants allows us to directly compare different states of supporting cell differentiation in the same tissue. In P0 mice, we saw a higher proportion of ectopic outer hair cells vs. inner hair cells in the apex of the cochlea, and an even smaller proportion of ectopic inner hair cells at the base. Since inner hair cells begin to differentiate before outer hair cells in any given region of the cochlea (Chen et al., 2002; Cai et al., 2013), it is possible that these differences reflect a neural-abneural gradient of response to Notch inhibition as well as an apical-basal response. Alternatively, since different supporting cell types express different combinations of *Hes* and *Hey* genes (Zheng et al., 2000; Zine et al., 2001; Hayashi et al., 2008; Li et al., 2008; Doetzlhofer et al., 2009; Murata et al., 2009; Tateya et al., 2011), it is possible that these differences reflect the different sensitivities of these Notch target genes to Notch inhibition (Ong et al., 2006).

We observe a down-regulation of mRNA levels of Notch receptors, ligands and downstream effectors in the first postnatal week. The degree and rate of down-regulation varies, but analysis of Notch1 signaling in cochlear supporting cells over this time period (Figure 5; Murata et al., 2006; Basch et al., 2011) suggests that very little cleavage of the Notch1 receptor is occurring by the end of the first postnatal week. The mechanism of this down-regulation is currently not known, although given the absence of significant Notch pathway expression in the adult cochlea, it is possible that the loci of Notch pathway genes are becoming epigenetically modified and placed beyond use. It is also possible that this epigenetic silencing is accompanied by silencing of the direct targets of Notch effectors such as the Hes and Hey genes. However, it should be noted that the Notch pathway appears to be down-regulated in mature supporting cells in the chicken basilar papilla, as this sensory organ also fails to respond to gamma secretase inhibitors in the undamaged state (Daudet et al., 2009). Nevertheless, after damage, the Notch pathway is once again deployed in chicken supporting cells and the differentiating hair cells that they generate (Stone and Rubel, 1999; Daudet et al., 2009). It will therefore be of great interest to identify the epigenetic state of Notch pathway genes and their targets in mature mammalian supporting cells. It should also be noted that the co-expression of Jag1 and Sox2, which is seen in sensory patches from their first appearance (Kiernan et al., 2005b; Pan et al., 2010, 2013; Neves et al., 2011, 2012), is maintained in adult mouse supporting cells (Oesterle et al., 2008). It is thus formally possible that low levels of Notch signaling may persist in the adult cochlea and may maintain expression of these two genes by lateral induction. If this is the case, such signaling does not appear to confer competence for regeneration on supporting cells.

A recent study demonstrated that a small but significant number of new hair cells could be generated from supporting cells by treating noise-damaged animals with gamma secretase inhibitors (Mizutari et al., 2013), leading to a partial restoration of function. How can we reconcile these results with our data in the present study? First, it is possible that the Notch pathway can continue to regulate hair cell and supporting cell fate in the adult animal when expressed at significantly lower levels. We feel this is unlikely since binding of the Notch-ICD-MAML-RBPj complex to its target sites in the genome is likely to be severely compromised at low concentrations of Notch-ICD (Ong et al., 2006). Second, it is possible that a sub-population of supporting cells continue to express Notch pathway components at significant levels, but that these would not be detected when analyzing gene expression in the entire cochlea. In our single cell analysis of 123 P21 pillar cells and Deiters’ cells, we were able to detect a very small number of cells in which Notch pathway components were expressed at comparable levels to their neonatal counterparts (Figure 6), and levels of *Hes5* and the hair cell marker *Atoh1* are more than 10-fold lower in the adult, even after noise damage (Figure 7). This suggests that if such cells persist in the adult cochlea, they are present in extremely small numbers. Finally, it is also possible that a second, Notch-independent pathway that can be targeted by gamma secretase inhibitors is operating in a small number of mature supporting cells. The effect of inhibiting this second pathway would be overshadowed by Notch inhibition in the neonatal cochlea, but might be uncovered in the adult cochlea when the Notch pathway is no longer active. It should also be noted that the noise damage protocol (Liu et al., 2011) used in our study—98 dB for 4 h—is significantly less severe than the protocol used by Mizutari et al. (116 dB for 2 h). However, the large and significant drop in *Atoh1* and *Hes5* levels we observe in undamaged adult tissue compared to neonatal animals still supports our observed down-regulation of Notch pathway genes in the first postnatal week.

In conclusion, our results suggest that the canonical Notch pathway is not active to any significant degree in the adult organ of Corti, and that the down-regulation of signaling occurs prior to the onset of hearing. The Notch pathway can therefore be viewed as a developmental scaffold for the organ of Corti—it is partly necessary for establishing the pattern and proportion of hair cells and supporting cells, but not necessary to maintain this pattern once it has been established. This suggests that inhibition of Notch signaling in the adult organ of Corti in the absence of other manipulations is unlikely to promote significant numbers of new hair cells, and that alternative or supplementary therapeutic interventions should be considered.

## Conflict of Interest Statement

The authors declare that the research was conducted in the absence of any commercial or financial relationships that could be construed as a potential conflict of interest.
